# Development of a passive doas system to retrieve atmospheric pollution columns in the 200 to 355 nm region

**DOI:** 10.1186/1735-2746-10-8

**Published:** 2013-01-08

**Authors:** Rubén Galicia Mejía, JoséManueldelaRosa Vázquez, Suren Stolik Isakina, Edgard Moreno García, Gustavo Sosa Iglesias

**Affiliations:** 1 SEPI-ESIME-Z. Instituto Politécnico Nacional, Av. IPN S/N, UPALM Edif Z, 3er piso cp., 07738, México, D.F., México; 2 Dirección de Investigación y Posgrado. Instituto Mexicano del Petroleo, Eje Cenetral Lázaro Cárdenas, México, D.F., México

**Keywords:** Spectroscopy, Pollution, Sulfur dioxide, Ozone, Ultraviolet

## Abstract

In recent years several techniques have been developed to measure and monitor the pollution of the air. Among these techniques, remote sensing using optical methods stands out due to several advantages for air quality control applications. A Passive Differential Optical Absorption Spectroscopy system that uses the ultraviolet region from 200 to 355 nm of the solar radiation is presented. The developed system is portable; therefore it is practical for real time and in situ measurements. The enhanced wavelength range of the system is intended to detect the ultraviolet light penetration in the Mexican Valley considering the solar zenith angle and the altitude. The system was applied to retrieve atmospheric SO_2_ columns emitted either by anthropogenic (power plant) or natural sources (volcano), reaching a detection limit of about 1 ppm. The measurement of the penetrating solar radiation on the earth surface at the UVC range is presented and the possibility to measure pollution traces of some contaminants as O_3_, NO_2_ and aromatic compounds in real time and in situ in the ultraviolet region is discussed.

## Introduction

The production of thousands of chemicals has contributed to industrial and economic development in many parts of the world. This trend however has been associated with the release of new chemicals and possibly toxic substances into the environment and food chain and adversely affecting human health in many instances. The pollutants associated with the anthropogenic activities could be inorganic as well organic compounds [1]. The air pollution is very complex dynamic phenomena. It could be associated with the industrial activities as well as with the concentration of the population in large towns and cities [2].

The power plants are using resources like fuel and water to provide electricity that is one of the essential needs for sustainable development and life. This activity produces and discharges all different kinds of pollutants such as, gaseous, liquid, electromagnetic fields, and noise which endanger our lives and environments. The amounts of pollutants discharged by power plants, especially air pollution are more than assimilation capacity of nature because now it is clear that sustaining and assimilative capacity of the biosphere though tremendous, is after all finite. The first step is to understand the magnitude of emissions from each source [3].

The Differential Optical Absorption Spectroscopy (DOAS) has been successfully used for air pollution detection in several research works [4]. Important advantages over other techniques like gas chromatography, chemiluminescence, and electrochemical, gravimetric, and matrix isolation, are the lower cost and the time saving, especially when the sunlight is used as the source (passive DOAS). This technique is particularly interesting in the monitoring of pollution emissions by refineries or thermoelectric power stations.

Due to the absorption by the O_3_ layer above the earth in the UVC interval (200 to 290 nm), the DOAS technique has been based on the UVB-VIS range absorption measurements. It has been used to detect chemicals such as CO, SO_2_, O_3_, NO_2_ HNO_2_, HCHO, CH_3_, CHO, benzene, toluene, p- and m-Xylene [5].

The absorption lines of these pollutants interfere with eachother making more laborious the processing of the measured spectra. Usually, the measurement of SO_2_ columns by the passive DOAS technique is done using the 290 to 330 nm wavelength range, which is well-known penetrates at sea level. In this region the SO_2_ absorption lines interfere with the absorption lines of less common species as HCHO, BrO, O_3_ and ClO [4].

In the wavelength range from 230 to 260 nm the ozone is the principal absorber. Benzene and toluene have cross sections one order of magnitude lower than O_3_[4]. Ozone atmospheric measurements have been previously reported using active DOAS in the range from 265 to 345 nm [6,7]. Also, atmospheric O_3_ measurements using passive DOAS have been done in the range from 438.5 to 540 nm. However due to the overlapping of its absorption spectrum with the NO_2_ absorption spectrum the concentration of both elements is measured [8].

It is very well documented that the solar radiation in the 190–280 nm range is strongly absorbed by the stratospheric ozone. Hitherto every report states that almost all incoming solar UVC and 90% of UVB are absorbed by stratospheric ozone [9]. As a result, there are no published experiments measuring the absorption levels in the UVC range. However, if there is any irradiance left, once the radiation passes to the troposphere the O_3_ concentration decreases and it would be possible to detect the remaining radiation in UVC with a sufficiently sensitive detector.

The solar irradiance at the earth’s surface varies greatly depending on factors such as latitude, time of the day, month of the year, cloud cover, and haze (aerosols). The spectral irradiance in the UVC range on the earth surface also depends on the ozone column, on the altitude above sea level and the solar zenith angle. Very sensitive measurements from 280 to 300 nm at sea level, 33^°^ North latitude and 25.14^°^ Solar Zenith Angle (SZA), using PMT detectors, reported the detection of solar spectral irradiance of 3x10^-4^ Wm^-2^ nm^-1^[10]. Similar measurements have reported a value of two orders of magnitude higher at 2500 m altitude, 22^0^ North latitude and 7.8^°^ SZA [11].

The Mexican Valley is a high plateau in central Mexico with a minimum altitude of 2200 m and latitudes between 19^o^ and 20^o^ North Latitude. Under such conditions the solar zenith angle is almost 0^o^ at noon. By what, these values propitiate a higher probability to detect some irradiance at UVC wavelength range with very sensitive detectors as photomultiplier tubes used in spectroradiometers or charged coupled devices (CCD) used in minispectrometers [12]. In Mexico City the O_3_ column amounts between 240 and 320 DU [13], if there is a possibility to detect some spectral irradiance in the UVC region it could be possible to detect traces absorbing in this wavelength interval. So, in order to prove the usefulness of the passive DOAS technique in the range from 200 to 350 nm in the Mexican Valley, we have developed a portable system to measure pollution slant columns in this spectral range of several pollutants as SO_2,_ O_3_, NO_2_, aromatic compounds, etc.

In this issue the SO_2_ emissions around the Tula's Industrial Complex in the Mexican Valley and around the Popocatepetl Volcano are reported. These calculations were performed using the developed system. Some discussion is presented regarding the observed well-structured absorption spectra in the range of 200 to 290 nm.

## Materials and methods

The system consists of a fused silica lenses telescope 84-UV-25 (Ocean Optics) with a 25.4 mm diameter, focal length of 100 mm. This telescope is especially suitable for light collimation from a distant source. The collimated light is guided to the spectrometer using an optical fiber (QP600-2-SR, Ocean Optics) with a 600 m diameter core and 2 m long. The spectra measurement was performed using a miniature spectrometer (USB 2000, Ocean Optics) with a diffraction grating of 2400 lines/mm. A cylindrical lens is incorporated to the detector to increase light collection efficiency focusing a 1 mm high slit onto a considerably shorter (200 μm) detector elements. The CCD detector (ILX511A, Sony) is coated with an UV anti-reflecting film to reduce the reflection losses and therefore increasing their sensitivity. The spectral range of the spectrometer is in the 190 to 355 nm range with a resolution of 0.5 nm. This system shows a high signal to dark current random noise ratio in the 190 to 290 nm range (over 3 times for measurements under pollution measurements and about 12 times under clean sky measurements). The system was checked and no overlapping of different diffraction orders and the scattering of the incident light which could affect the readings in the UVC region was detected. To reduce the background noise of the measurement an averaging of 3 to 5 spectra was performed.

The coordinates of the measuring spots were determined with a GPS locator (GT 37231). All the information inputs a laptop were the developed software processes the data. The coordinates given by the GPS locator were superimposed on the Google satellite images using the Google-Earth-KH-80.llb libraries to show the actual measuring points on a satellite photograph.

The attenuation of the irradiance *I*_0_(*λ*) is described by the Beer-Lambert’s Law *I*(*λ*) = *I*_0_(*λ*)*e*^− *σ*(*λ*) ∫ *c*(*s*)*ds*^. The attenuation depends of the concentration *c*(*s*) of the different compounds of the light path media [14]. The absorption cross section *σ*(*λ*) defines the light absorption to a specific wavelength. The dependence of the cross sections *σ*(*λ*)on the temperature and pressure has not been considered in this work. The gases columns are evaluated by using the mixing ratio method [15]. The light absorption cross section of a specific pollutant can be obtained from reported data on literature or from laboratory measurements.

Usually, it is very difficult to obtain separately the pollutant concentrations *c*(*s*) and the optical length *l*=∫*ds*. Therefore, these parameters are combined to form what is called slant column density *S*=∫*c*(*s*)*ds* expressed in molecules/cm^2^[16,17].

Actually, the measured absorption involves all the present molecules that absorb in that specific wavelength. The absorption of several substances *i* will be then described by the following expression: $I\left(\lambda \right)=I_{0}\left(\lambda \right)e^{-\sum_{i}\sigma_{i}\left(\lambda \right)S_{i}}$, where *σ*_*i*_(*λ*), *c*_*i*_(*S*) and *S*_*i*_ are their absorption cross section, its specific concentration and its slant column density, respectively. The sunlight spectrum that reaches the Earth surface shows slow and fast variations. The slow variations of the spectrum are determined by the light source and by the scattering. The fast variations are determined by the absorption of molecules. The spectrum of the cross sections can be divided in a slow variations *σ*_*i*_^*S*^(*λ*) and fast variations parts *σ*_*i*_^*F*^(*λ*). Therefore, the absorption cross section for each component will be *σ*_*i*_(*λ*) = *σ*_*i*_^*S*^(*λ*) + *σ*_*i*_^*F*^(*λ*).

The slow variations part of the measured light intensity, which depends on *I*_0_(*λ*), can be approximated by a polynomial. Using some polynomial adjustment method or by using high-pass digital filters, for instance the Savitzky-Golay [18] a solution for fast variations part *σ*_*i*_^*F*^(*λ*) could be obtained [4,19].

For the detection of only one gas component in a spectral interval, if the scattering is small compared to the slow variations of radiation *I*_0_(*λ*) the pollutant slant column density can be expressed as:

$$S=\frac{\text{ln}\left(\frac{I_{0}\left(\lambda \right)}{I\left(\lambda \right)}\right)}{\sigma^{F}\left(\lambda \right)}=\frac{D\left(\lambda \right)}{\sigma^{F}\left(\lambda \right)}$$

Where *D*(*λ*) is the Optical Density of the medium [4,15]. The cross sections of the gas trace at the atmosphere could be considered independent from their placement; this means that they are independent from their altitude [20].

High resolution absorption spectrum for the SO_2_ has been reported [21]. The reference spectrum for the evaluation of the measurements is needed and it must consider only the fast variations *σ*_*i*_^*F*^(*λ*) and the spectral deformations produced by the spectrometer and determined by the spectrometer transfer function (STF). In practice, to obtain the STF the spectral lines emitted by a Hg lamp could be used because, these are narrower (~10 pm) [22] than the typical resolution of the used spectrometer (0.1–1 nm). The measured spectrum is a good approximation of the spectrometer transference function [17]. Mathematically the measured spectrum I^*^(*λ*) can be expressed as the convolution of the Hg lamp emission spectrum I(*λ'*) with the spectrometer transference function I*(*λ*) = *I*(*λ*^′^)  *H*_*sp*_(*λ*^′^), where *H*_*sp*_(*λ'*) is the spectrometer transfer function. The STF was measured at 334.1 nm because at this wavelength the “cleanest” signal is obtained.

**Figure 1:**
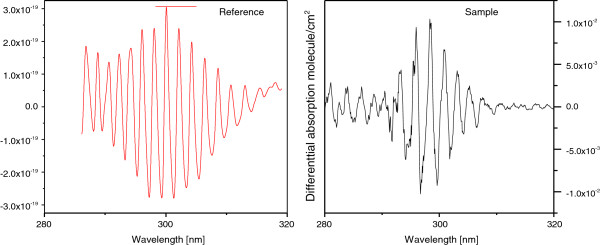
**Reference and Sample spectra to determine the SO**_**2**_**content.**

DOASIS software, developed at Heidelberg University [23], was used to perform the convolution of the transference function of our instrument. The high resolution SO_2_ spectrum is used to obtain our reference spectrum. To observe if there is any shift of the SO_2_ reference spectrum related to the convolution process, the SO_2_ absorption spectrum with a Xe lamp (Newport, model 969607) was measured. A constant shift of 0.98 nm was obtained and it must be considered when the trace gas concentration calculation is performed. Figure 1 shows the SO2 reference and sample spectra.

A 2.5 mbar, 3 cm long, SO2 cell was used to validate our DOAS system. We used a Xe-lamp as radiation source. The measured column agrees within a +− 4% error. To determine the density of a measured column a non-linear adjustment is done by using the Levenberg-Marquardt method [24] using the measured *I*_0_(*λ*) and the reference spectra *I*_0_(*λ*). A dark spectrum, previously captured, is subtracted from *I*(*λ*) and *I*_0_(*λ*). A residual Δ*ψ* is obtained from the fitting process, in our case in the order of 2.5x10^17^ particles/cm^2^, which can be used to calculate the detection limit of our system trough the relation $S_{\min}=\frac{\Delta \psi }{\sigma^{'}}$[17]; hence the detection limit is the order of 1 ppm.

The spectrometer is connected to a laptop. A program was developed in Labview to capture and filter the measured spectra to perform the non-linear adjustment and to identify the amount of polluting gas. This software consists of several programming stages. First of all, a driver for the Windows operative system is installed from the spectrometer that comes from the OOIDDrv32.dll (Ocean Optics, 2011) library. Once the hardware is recognized a Sarvitzky-Golay filter program filters the reference *I*_0_(*λ*) and measured *I*(*λ*) spectra. A dark spectrum is subtracted from and *I*(*λ*) to reduce the thermal noise of the instrument itself. Immediately, the relation of these spectra is calculated and is applied the logarithm function. Spectra are geographically referenced throughout the journey to integrate all the SO_2_ measured columns. A mouse-type GPS was used with the NMEA protocol to link it with the program, to do so, an API for Windows is used which is provided by Labview under the VISA name, which allows to read the data from the GPS ([25]; Visa, [26]).

When the DOAS technique is used, the path length of light depends on the number of scattering events that take place until the photon reaches the spectrometer. Some methods to obtain at least the statistics of photon propagation are based on the simulation Monte Carlo method [17] to be able to validate statistically what happens with a large amount of photons it is necessary to simulate such events unfortunately this process requires a computing robust system and the simulation process is not performed in real time. That is not practical for insitu measurements. Taking this into consideration, in this work the column densities are indicated in ppm*m, using mixing ratios [27,28]. The SO_2_ flow is calculated in tones by day [14], using column densities measured by the system, which must be multiplied by the distance of the traverse route using the following formula: $\mathrm{ton}/\mathrm{day}=0.00023\int_{x_{1}}^{x_{n}}SO_{2}\left(\mathrm{ppm}\right)*X*\mathrm{Vsen\theta}\mathrm{dX}$ Here *X* is the traverse distance in meters from the point *x*_*1*_ to *x*_*n*_, *V* is the wind speed in m/s, *θ* is the angle between vectors *X* and *V*. For the real time in situ measurements around the industrial zone and the volcano, the telescope was fixed on a car for the traverse. Five spectra were averaged in each data entry to minimize the noise effects. The spectra are taken every 3 seconds. The system takes firstly the dark spectrum to reduce the noise produced by the spectrometer and the skylight spectrum *I*_*skl*_(*λ*) is measured. Finally, the sample spectra are taken during the traverse around the industrial zone.

## Results

The reference and measured spectra used for the determination of SO_2_ content in the 280–320 nm wavelength range are shown in Figure 1. The reference spectrum was obtained according to the method described earlier. The absorption bands of SO_2_ are very well defined in this interval.

**Figure 2:**
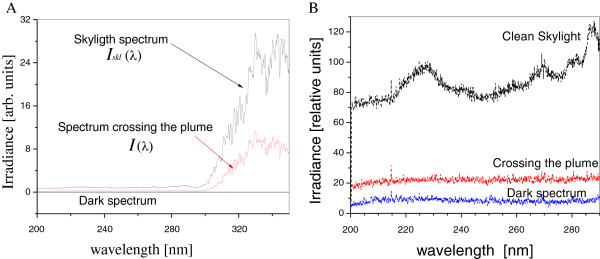
**A. clean skylight spectrum *I***_***skl***_**(*λ*), spectrum while crossing the plume *I*(*λ*), and the dark spectrum in the whole working wavelength range.B**. Same optical Irradiance spectra enlarged in the UVC wavelength interval.

The optical spectra in the 200-350 nm wavelength interval with and without contamination are presented in Figure 2A. The skylight spectrum is detected with a “clear” sky, which means, without contamination plume. It is clearly observed the attenuation of the spectral irradiance on the Earth surface due to the pollutants absorption. More than that, it is perfectly observed that a certain “non despicable” irradiance is detected in the UVC region. The UVC wavelength interval has been enlarged and presented in Figure 2B where an absorption band structure is observable. The attenuation in the plume in the range between 200 to 290 nm of the measured spectrum *I*(*λ*) could be related to the O_3_ concentration, which is the main atmosphere component absorbing in that region.

**Figure 3:**
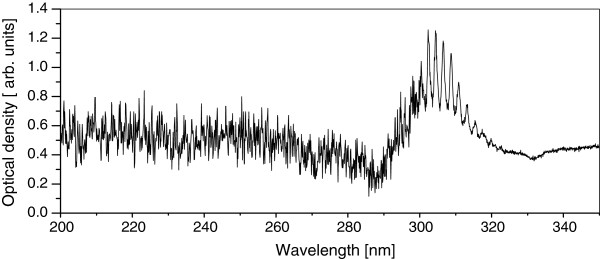
Optical density observed from 200 to 350 nm.

The optical density is presented in Figure 3. The characteristic SO_2_ absorption bands are very well defined in the spectrum range between 290 and 320 nm. Besides, a band structure related to scattering and absorption in the presence of other molecules in the UVC range can be observed. Usually, this interval is neglected in most studies. This result shows the importance to develop some similar algorithms to evaluate the concentration of species absorbing in this range, such as tropospheric ozone.

**Figure 4:**
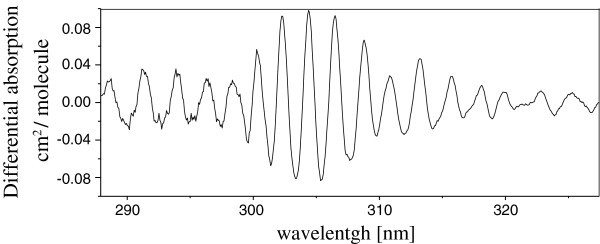
**SO**_**2**_**differential absorption spectrum in the 290 to 320 nm interval after filtering** .

After the high-pass filter is applied and the spectrum restricted from 290 to 315 nm range, the differential absorption SO_2_ spectrum is obtained and it is presented in Figure 4.

The developed system was used to measure both, anthropogenic and natural SO_2_ emissions. Two sites with significant SO_2_ concentration were chosen near Mexico City.

### Measurements at Tula hidalgo refinery

**Figure 5:**
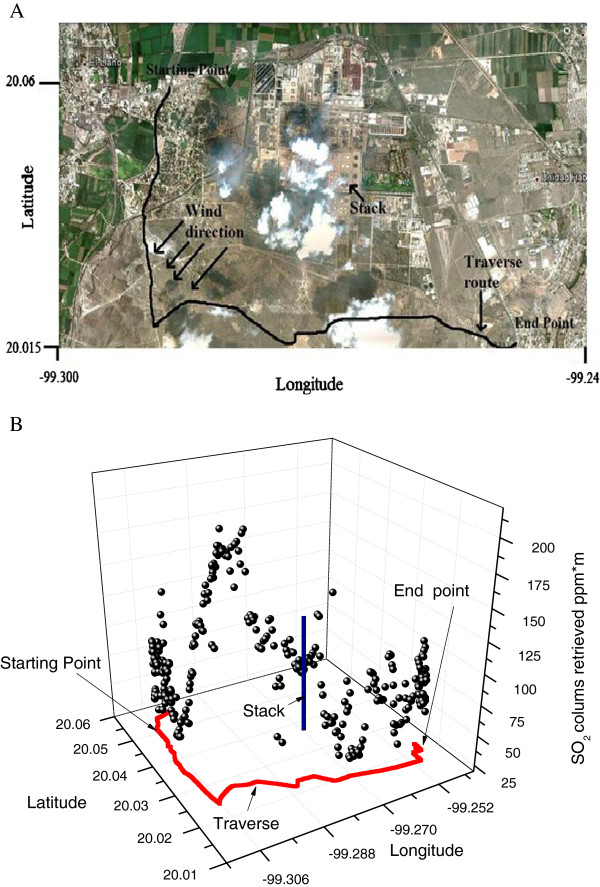
**A. Satellite image from Miguel Hidalgo refinery in Tula. The SO**_**2**_**polluting chimney, the wind direction, and the traverse route are presented.B.** shows SO_2_ measured columns with passive DOAS system around Miguel Hidalgo PEMEX refinery in Tula Hidalgo.

In Tula Hidalgo, Mexico, two nearby industries are located: “Miguel Hidalgo” PEMEX oil crude refinery and CFE thermo power station “Francisco Pérez Ríos”. The satellite image is presented in Figure 5A. Once the traverse was completed, the SO_2_ columns are calculated using a wavelength interval from 290–350 nm. The variations in ppm*m can be observed in each column and the results are shown in Figure 5B. Both industries are responsible for the emission of nearly 350 t/d of SO_2_[29]. In this sense, it is a convenient place to test the equipment. The wind direction is presented on the satellite image, Figure 5A. Also, the traverse route is shown on both, the satellite image (Figure 5A) and the corresponding SO_2_ column distribution (Figure 5B). The starting and ending points of the traverse are indicated. The result presented in Figure 5 (A and B) corroborates that the distribution of the SO_2_ columns with higher concentrations are related directly with the wind direction.

### Measurements at Popocatepetl volcano

**Figure 6:**
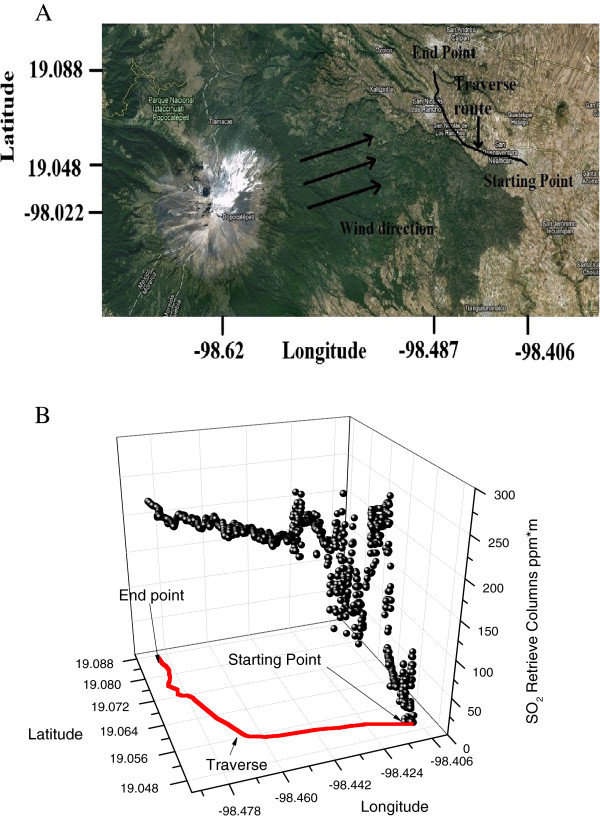
**A. Satellite image from Popocatepetl volcano in which the wind direction and the traverse route are depicted.B**. shows SO_2_ measured columns at Popocatepetl volcano with passive DOAS system.

Popocatepetl volcano is located at the boarding limits between Estado de Mexico, Puebla and Morelos. Its coordinates are 19.023° North Latitude and 98.627° West Longitude. A traverse from San Buenaventura Nealtican to San Nicolas de los Ranchos in Puebla was done; it is shown in Figure 6A. It has to be noticed that the traverse direction is from east to west, as the starting and ending points are indicated. The measured SO_2_ columns are shown in Figure 6B. The first part of the traverse is oriented almost radially to the volcano coordinates. At the starting point the system was calibrated. As the measuring point approaches the volcano the SO_2_ columns readings increase, as it is expected due to the diffusion and the dilution of the pollution cloud.

At some point of the traverse, indicated with an arrow on Figure 6A, the direction changes and it becomes nearly perpendicular to the volcano’s direction. In this second part of the traverse the measured values of SO_2_ columns remain practically constant.

## Discussion

Usually, the measurement of SO_2_ columns by the passive DOAS technique is done using the 290 to 330 nm wavelength range. In the wavelength range from 230 to 260 nm the ozone is the principal absorber. Albeit the arrangement of our spectrometer shows less resolution than a similar one with a range from 290 to 355 nm, from Figures 5 and 6 it can be clearly observed that the developed system has still enough resolution to determine the SO_2_ pollutant distribution and, after Figure 3 (for altitude and latitude conditions as in the Mexican Valley), also could be used to simultaneously measure O_3_ columns in the 230 to 260 nm range.

Still is necessary the measurement of the wind direction and speed in the plume at the same time with the column measurement to estimate the flow of the SO_2_; this is an open problem. If the wind conditions remain steady as the columns measurements along an appropriate traverse are performed, a good approximation of the emitted concentration and the pollutant flow could be calculated.

## Conclusion

In this work we describe a passive DOAS system developed to determine SO_2_ polluting columns. Portability of the developed system makes it practical for real time measurements and it allows the SO_2_ columns measurements in situ*.* It could be applied to develop some models of pollutants distribution considering the source, the diffusion and the wind conditions. The direct measurement of the pollutant columns provides useful information to evaluate the pollution level in real time. Also, DOAS technique avoids contact with the samples providing a better interpretation of the SO_2_ columns measurements.

Attenuation in the plume of the reference spectrum *I*_0_(*λ*) in the wavelength interval from 200 to 290 nm is presented in Figure 2B. It should be emphasized that a certain level of irradiance in the UVC region is detected on the earth surface. It could be related to the altitude and latitude of Mexican Valley, as well as with the continuous improvement of optical detectors. The absorption in the UVC range could be related to the O_3_ concentration. It leads to a possibility to perform DOAS in this wavelength band applied to ozone detection and monitoring at Mexico City region, due to its altitude and latitude. However, much more research work should be done to corroborate this hypothesis.

## Competing interests

The authors declare that they have no competing interests.

## Authors’ contributions

RGM, EMG and GSI have implemented the DOAS system and have made substantial contribution to acquisition of data. RGM, SSI and JMRV have made substantial contribution to the calibration of the system interpretation of data and in drafting the manuscript. RGM,SSI, JMRV, GSI read and approved the final manuscript. All authors read and approved the final manuscript.
